# Notice to Readers: *MMWR *Weekly Launches Online Manuscript Submission System

**DOI:** 10.15585/mmwr.mm6601a9

**Published:** 2017-01-13

The* MMWR* Weekly is now using *MMWR* ScholarOne Manuscripts, an online system for manuscript submissions. Launching this system provides comprehensive workflow management and streamlines the Weekly submission process.

Using *MMWR* ScholarOne Manuscripts allows manuscripts to be transmitted electronically; makes manuscript files accessible to editors through the submission site; and enables authors to check the status of manuscripts and update contact information.

All manuscripts for the Weekly must be submitted through *MMWR* ScholarOne Manuscripts at https://mc.manuscriptcentral.com/mmwr. Manuscript and proposal submission for the Serials (i.e., Recommendations and Reports, Surveillance Summaries, and Supplements) should continue to be submitted by email to DCJohnson@cdc.gov.

Additional information on how to submit to the Weekly through *MMWR* ScholarOne Manuscripts is available at https://www.cdc.gov/mmwr/submissions.html.

